# Supplementary material for: VBCG: 20 validated bacterial core genes for phylogenomic analysis with high fidelity and resolution
Source: Microbiome. 2023 Nov 8;11:247. doi: 10.1186/s40168-023-01705-9 (PMC10631056; doi:10.1186/s40168-023-01705-9)
Supplement: Supplementary file 2 — Additional file 1. [file 40168_2023_1705_MOESM1_ESM.docx]

**Supplementary materials**

**Table S1.** The 85 core genes with both presence and single-copy ratios > 95%. The presence and single-copy ratios were calculated based on the 5506 representative genomes.

| HMM | Presence ratio | Single-copy ratio | Product |
| --- | --- | --- | --- |
| TIGR01050.1 | 99.6% | 99.6% | 30S ribosomal protein S19 |
| PF00380.22 | 99.5% | 99.4% | Ribosomal protein S9/S16 |
| TIGR01067.1 | 99.5% | 99.5% | 50S ribosomal protein L14 |
| TIGR00981.1 | 99.4% | 99.4% | 30S ribosomal protein S12 |
| PF00281.22 | 99.4% | 99.4% | Ribosomal protein L5 |
| PF00410.22 | 99.4% | 99.4% | Ribosomal protein S8 |
| TIGR01049.1 | 99.4% | 99.3% | 30S ribosomal protein S10 |
| TIGR01164.1 | 99.4% | 99.4% | 50S ribosomal protein L16 |
| PF00276.23 | 99.3% | 99.3% | Ribosomal protein L23 |
| TIGR01029.1 | 99.3% | 99.2% | 30S ribosomal protein S7 |
| TIGR00952.1 | 99.3% | 99.2% | 30S ribosomal protein S15 |
| TIGR00029.2 | 99.3% | 99.2% | 30S ribosomal protein S20 |
| TIGR03631.1 | 99.2% | 99.2% | 30S ribosomal protein S13 |
| TIGR00060.1 | 99.1% | 99.0% | 50S ribosomal protein L18 |
| TIGR01066.1 | 99.1% | 99.0% | 50S ribosomal protein L13 |
| TIGR00855.1 | 99.1% | 98.8% | 50S ribosomal protein L7/L12 |
| TIGR03654.1 | 99.1% | 99.1% | 50S ribosomal protein L6 |
| PF00466.23 | 99.0% | 98.9% | Ribosomal protein L10 |
| TIGR01171.1 | 99.0% | 99.0% | 50S ribosomal protein L2 |
| TIGR01021.1 | 98.9% | 98.9% | 30S ribosomal protein S5 |
| TIGR01032.1 | 98.9% | 98.8% | 50S ribosomal protein L20 |
| TIGR00001.1 | 98.9% | 98.9% | 50S ribosomal protein L35 |
| TIGR02027.1 | 98.9% | 98.1% | DNA-directed RNA polymerase subunit alpha |
| TIGR01071.1 | 98.9% | 98.9% | 50S ribosomal protein L15 |
| TIGR03953.1 | 98.8% | 98.7% | 50S ribosomal protein L4 |
| TIGR03632.1 | 98.8% | 98.7% | 30S ribosomal protein S11 |
| TIGR00062.1 | 98.7% | 98.7% | 50S ribosomal protein L27 |
| TIGR00116.1 | 98.7% | 98.5% | translation elongation factor Ts |
| TIGR00166.1 | 98.6% | 98.5% | 30S ribosomal protein S6 |
| TIGR01169.1 | 98.6% | 98.6% | 50S ribosomal protein L1 |
| TIGR01017.1 | 98.6% | 95.7% | 30S ribosomal protein S4 |
| TIGR00086.1 | 98.5% | 97.9% | SsrA-binding protein |
| TIGR00158.1 | 98.5% | 98.4% | 50S ribosomal protein L9 |
| TIGR03625.1 | 98.4% | 98.4% | 50S ribosomal protein L3 |
| TIGR01079.1 | 98.3% | 98.3% | 50S ribosomal protein L24 |
| TIGR00967.1 | 98.3% | 97.7% | preprotein translocase subunit SecY |
| TIGR00043.2 | 98.3% | 98.2% | rRNA maturation RNase YbeY |
| TIGR00061.1 | 98.3% | 98.2% | 50S ribosomal protein L21 |
| TIGR01632.1 | 98.3% | 98.1% | 50S ribosomal protein L11 |
| TIGR00414.1 | 98.2% | 95.6% | serine--tRNA ligase |
| TIGR01024.1 | 98.2% | 98.2% | 50S ribosomal protein L19 |
| TIGR01030.1 | 98.1% | 98.1% | 50S ribosomal protein L34 |
| TIGR00006.1 | 98.1% | 96.4% | 16S rRNA (cytosine(1402)-N(4))-methyltransferase RsmH |
| TIGR00168.2 | 98.1% | 95.3% | translation initiation factor IF-3 |
| TIGR00755.1 | 98.0% | 98.0% | 16S rRNA (adenine(1518)-N(6)/adenine(1519)-N(6))-dimethyltransferase RsmA |
| TIGR03635.1 | 98.0% | 98.0% | 30S ribosomal protein S17 |
| TIGR00468.1 | 98.0% | 97.9% | phenylalanine--tRNA ligase subunit alpha |
| TIGR01011.1 | 98.0% | 97.9% | 30S ribosomal protein S2 |
| TIGR01044.1 | 97.8% | 97.8% | 50S ribosomal protein L22 |
| TIGR00396.1 | 97.8% | 97.4% | leucine--tRNA ligase |
| TIGR03594.1 | 97.7% | 97.6% | ribosome biogenesis GTPase Der |
| TIGR00496.1 | 97.7% | 97.6% | ribosome recycling factor |
| TIGR00392.1 | 97.6% | 95.8% | isoleucine--tRNA ligase |
| TIGR00442.1 | 97.6% | 95.2% | histidine--tRNA ligase |
| TIGR03723.1 | 97.5% | 96.6% | tRNA (adenosine(37)-N6)-threonylcarbamoyltransferase complex transferase subunit TsaD |
| TIGR00964.1 | 97.5% | 97.4% | preprotein translocase subunit SecE |
| PF00162.22 | 97.5% | 95.8% | Phosphoglycerate kinase |
| TIGR02013.1 | 97.4% | 97.1% | DNA-directed RNA polymerase subunit beta |
| TIGR02729.1 | 97.3% | 97.3% | Obg family GTPase CgtA |
| TIGR00019.1 | 97.3% | 97.3% | peptide chain release factor 1 |
| TIGR00810.1 | 97.2% | 97.0% | preprotein translocase subunit SecG |
| TIGR00092.1 | 97.2% | 97.1% | redox-regulated ATPase YchF |
| TIGR00012.1 | 97.2% | 97.2% | 50S ribosomal protein L29 |
| TIGR01393.1 | 97.2% | 96.3% | translation elongation factor 4 |
| TIGR01009.1 | 97.1% | 97.1% | 30S ribosomal protein S3 |
| TIGR00344.1 | 97.1% | 96.4% | alanine--tRNA ligase |
| TIGR00059.1 | 97.0% | 96.9% | 50S ribosomal protein L17 |
| TIGR02432.1 | 97.0% | 96.9% | tRNA lysidine(34) synthetase TilS |
| TIGR01391.1 | 96.9% | 95.7% | DNA primase |
| TIGR01953.1 | 96.8% | 96.8% | transcription termination factor NusA |
| TIGR02075.1 | 96.7% | 96.1% | UMP kinase |
| TIGR00002.1 | 96.7% | 96.6% | 30S ribosomal protein S16 |
| TIGR00088.1 | 96.6% | 96.6% | tRNA (guanosine(37)-N1)-methyltransferase TrmD |
| TIGR00487.1 | 96.5% | 96.4% | translation initiation factor IF-2 |
| TIGR00959.1 | 96.4% | 96.4% | signal recognition particle protein |
| TIGR00431.1 | 96.2% | 96.1% | tRNA pseudouridine(55) synthase TruB |
| TIGR03263.1 | 96.1% | 96.0% | guanylate kinase |
| TIGR00922.1 | 96.1% | 96.1% | transcription termination/antitermination protein NusG |
| TIGR00020.2 | 95.9% | 95.8% | peptide chain release factor 2 |
| TIGR00064.1 | 95.7% | 95.7% | signal recognition particle-docking protein FtsY |
| TIGR00472.1 | 95.7% | 95.6% | phenylalanine--tRNA ligase subunit beta |
| TIGR00460.1 | 95.5% | 95.3% | methionyl-tRNA formyltransferase |
| TIGR00152.1 | 95.4% | 95.1% | dephospho-CoA kinase |
| TIGR00250.1 | 95.3% | 95.2% | Holliday junction resolvase RuvX |
| TIGR00635.1 | 95.2% | 95.1% | Holliday junction branch migration DNA helicase RuvB |

**Table S2.** The 20 validated bacterial core genes upon the screens based on presence ratio, single copy ratio, and phylogenetic fidelity. The HMM ID of the genes, Robinson-Foulds (RF) distances from the corresponding *16S rRNA* gene trees and the products of the genes were listed.

| Rank | Core genes | RF Mean | RF STD | Products |
| --- | --- | --- | --- | --- |
| 1 | TIGR02013.1 | 115.9 | 14.8 | DNA-directed RNA polymerase subunit beta |
| 2 | TIGR03594.1 | 120.1 | 13.4 | ribosome biogenesis GTPase Der |
| 3 | TIGR01171.1 | 120.3 | 9.9 | 50S ribosomal protein L2 |
| 4 | PF00281.22 | 120.3 | 11.2 | Ribosomal protein L5 |
| 5 | TIGR02027.1 | 121.8 | 11.4 | DNA-directed RNA polymerase subunit alpha |
| 6 | TIGR01011.1 | 122.0 | 12.0 | 30S ribosomal protein S2 |
| 7 | TIGR01009.1 | 122.3 | 10.3 | 30S ribosomal protein S3 |
| 8 | TIGR00959.1 | 123.2 | 11.9 | signal recognition particle protein |
| 9 | TIGR00967.1 | 123.9 | 11.6 | preprotein translocase subunit SecY |
| 10 | TIGR01393.1 | 124.1 | 13.8 | translation elongation factor 4 |
| 11 | TIGR00487.1 | 124.1 | 12.3 | translation initiation factor IF-2 |
| 12 | TIGR01029.1 | 125.3 | 10.9 | 30S ribosomal protein S7 |
| 13 | TIGR01021.1 | 125.8 | 11.4 | 30S ribosomal protein S5 |
| 14 | TIGR00468.1 | 126.5 | 11.7 | phenylalanine--tRNA ligase subunit alpha |
| 15 | TIGR00019.1 | 126.6 | 10.8 | peptide chain release factor 1 |
| 16 | TIGR01169.1 | 126.7 | 14.0 | 50S ribosomal protein L1 |
| 17 | TIGR01953.1 | 127.6 | 12.3 | transcription termination factor NusA |
| 18 | TIGR03625.1 | 127.8 | 12.4 | 50S ribosomal protein L3 |
| 19 | TIGR02729.1 | 128.7 | 11.7 | Obg family GTPase CgtA |
| 20 | TIGR00092.1 | 128.8 | 12.1 | redox-regulated ATPase YchF |

**
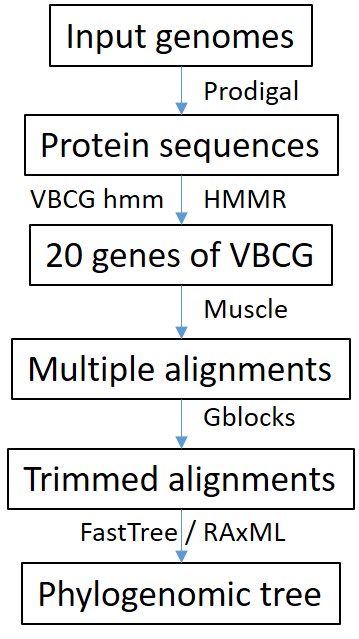
**

**Figure S1.** The workflow of the pipeline VBCG. The pipeline begins by predicting gene and protein sequences of the input genomes using Prodigal. Next, the protein sequences are used to identify the 20 VBCG genes with HMMER. The resulting VBCG genes of all the genomes are retrieved and put into separate files for each gene. The genes are aligned using Muscle, and any terminal gaps are removed from the multiple alignments. To select conserved blocks, Gblocks is applied. The processed alignments of the core genes are then concatenated, removing any taxa with more than one gene missing. Finally, the concatenated alignments are converted into Phylip files and fed to FastTree or RAxML for phylogenetic tree reconstruction.


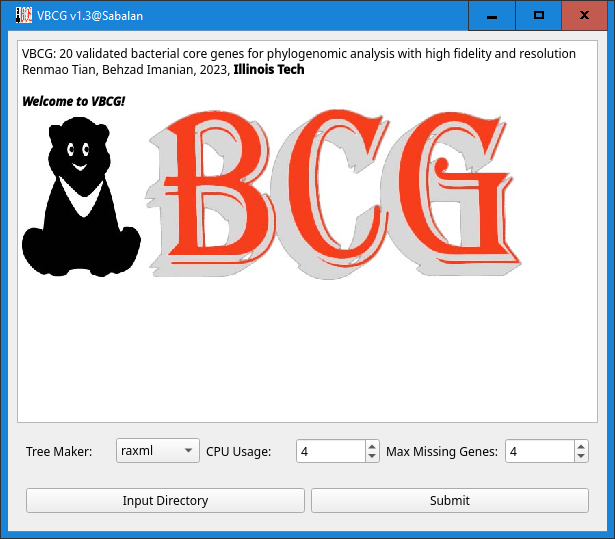


**Figure S2.** The interface of the VBCG desktop app (Windows version). Users only need to input genomic sequences and set the parameters, and it will identify the 20 VBCG genes with HMMER, and use the concatenated sequence for phylogenomic tree construction.
